# Present College Session

**Published:** 1856-01

**Authors:** 


PRESENT COLLEGE SESSION.

There is at present in attendance, the largest class ever assembled in our halls since the organization of this College; there being between seventy and eighty matriculants. The record book is not at hand, and therefore we do not speak precisely.

Several of our eastern States are represented in the class, as Connecticut, Pennsylvania, Virginia, Ohio, Indiana, Kentucky, Missouri, Illinois, Michigan; with the largest number from our own State.

The members of the class manifest a spirit of emulation and enthusiasm, in the progress of their studies, most gratifying to the faculty, and which gives assurance that they will advance to usefulness and efficiency.

The institution has reached beyond the days of its hardest trials. It is now a "fixed fact," and we would say to our co-laborers in the work of medical instruction that our efforts to advance it to this prosperous state, like many others which have heretofore sprung up, and whose projectors and friends are now enjoying the fruits of their efforts in prosperity and success. We have also had our tribulations and trials to meet and overcome.

In these struggles we have based our defence upon the eternal principles of truth and justice, and in this crucible we are willing that our acts shall be analyzed, and we challenge the investigation. We have taken open grounds, and prefer the open day contest to the covert and undercurrent efforts of those who have predicted our downfall, and have resorted to *every low measure* which could originate in a mean and craven spirit, in order to the accomplishment of our destruction. The dark spirit who has been the leader in these hostile attacks may now draw in its "Janus face," provided such a sentiment as that of shame ever was, or ever could be entertained by it.

From the few others engaged, and who, by false representations, were induced indirectly to aid in the destruction of a State enterprise in their midst, we did expect other and better things.

Religious prejudices were attempted to be awakened, and the power of a secret combination was also directed against us. Into an assemblage of the ministers of a strong and popular denomination a *bomb* was thrown, which was intended to explode to our destruction, in order, finally, to obtain their endorsement of an Eutopian scheme, but to succeed with this, there must first be a prejudice raised against us. How inconsistent to ask the endorsement of a religious sect, when the spirit at the head of the opposition, was one of the leaders of the Abner Kneeland sect of philosophers, and had prepared an address to be delivered on the occasion of the anniversary of the birth day of Thomas Paine. Let a denial ot this be made, and we are ready with the written proof, and yet this religious assembly did actually enter into measures for adopting this scheme as a part of an institution of that particular sect. This was doubtless done, ignorant of all the facts in the case, and to which we are not disposed in the least to enter our protest. We leave this matter to be adjusted by themselves, between themselves. The attack made upon us was without foundation, and the individual member of the Faculty implicated, and residing at a distance, is prepared to disprove the whole charge. A more unjust attack never was made upon an institution, but although the individual who made it may have been ignorant of the purpose or the object of it, yet it was plain that there was a "power behind the throne" in this matter, whose design was first to injure the State institution, and then to secure to another enterprise the protecting ægis of a strong religious denomination, in whose tenets and doctrines—nor the tenets or doctrines of any other religious creed the spirit at the bottom of it, did not believe *one word*. But this is only one of the *bold* efforts which have been resorted to to prostrate and destroy a State institution.

To those who have acted as became the friends of scientific enterprises, and especially when struggling into efficiency and usefulness in their midst, we say to them that they will be remembered with gratitude for their liberal and magnanimous conduct toward us when traduced and assailed by those who, disregarding every principle of right and honor, falsely and maliciously persisted in assailing us in every quarter.

We are superior to such attacks, as we are above the practice of such low measures, and we *defy* such efforts, as we despise the base, malignant, and corrupt source whence it all emanated.

